# Book Review: Complexity Perspectives on Researching Language Learner and Teacher Psychology

**DOI:** 10.3389/fpsyg.2021.720483

**Published:** 2021-07-29

**Authors:** Chao Zhang, Ruixi Zhang

**Affiliations:** School of Journalism and Information Communication, Huazhong University of Science and Technology, Wuhan, China

**Keywords:** complexity, language, language learner, psychology, teachers, interaction

As a paradigm, complexity offers interesting and innovative directions to scrutinize the idiosyncratic, interconnected, and emergent nature of the myriads of socioemotional and psychodynamic factors that play a key role in the process of language learning and teaching. To capture this dynamicity, complexity dynamic system theory (CDST) has exponentially gained interest in fields of second and foreign language education, in general, and language learner and teacher psychology, in particular, mainly at the theoretical level. However, more tangible examples are required to apply these theoretical ideas to methodological practice. To bridge these gaps, *Complexity Perspectives on Researching Language Learner and Teacher Psychology*, edited by Richard J. Sampson and Richard S. Pinner, aims to reconcile theory and practice by including theoretically illuminating and empirically robust studies that delve into various language learner and teacher psychology foci.

This volume consists of 16 chapters. In the introductory chapter, the editors foreground the significance of complexity perspectives on language learner and teacher psychology and succinctly outline the synopsis of the book. Drawing on the tenents of CDST, the authors in Chapter 2 thoroughly elaborate on such concepts as timescales, openness, predictability, variability, self-organization, attractor, and repeller states, as well as fractals, which can provide us with a fruitful glossary of these germane concepts to CDST. The author in Chapter 3 reports on how emotions can dynamically vary and develop and concludes that “multiple threading and timescales analysis does a reasonable job of furnishing visual representations of the emotional context that any teacher encounters and co-forms together with learners in a classroom” (p. 48). Embarking on the scenario-based method, narratives, and affective strategies, Chapter 4 presents the *Managing Your Emotions* questionnaire by taking into consideration the ecologies of language learning and teaching.

Utilizing a mixed-methods design, Chapter 5 provides evidence for the complexity and dynamicity of L2 willingness to communicate (WTC), concluding that “While traditional research helps us view phenomena clearly by simplifying them, a complexity perspective helps us capture complexity by closely examining it, but without necessarily simplifying it” (p. 83). In another evidence-based and mixed-methods study, Chapter 6, set in the Japanese context, delves into the complex issue of silence, a very underappreciated, yet influential topic, in the foreign language classroom. The authors conclude “the pairing of empirical data with an open-complexity approach showed how multiple, interconnected variables emerge over time to influence both individual and classroom silence” (pp. 99–100). Enlightened by his narratives of researching lifetime about the complexity, the author, in Chapter 7, features the notion of motivational resonance concerning learner self-concepts, educational cultures, and learner groups and recommends that teachers need to do action-based research. Set in the UK context, from the ecological perspective, Chapter 8 scrutinizes L2 motivation through exploratory practice with pre-sessional courses. The chapter highlights “the interconnectedness between students' life capital, life story and motivation” (p. 134). Chapter 9 opens up another intriguing dimension of the CDST by nullifying the reductionist approaches that try to compartmentalize the “messy” intricate essence of authentic classroom research and advocating the horticultural metaphor to contextualize dimensions of learner psychology in a listening class.

Embarking on Trajectory Equifinality Approach, Chapter 10 delineates how we can probe into the complex and dynamic learning trajectories of L2 learners and teachers, mediated by socio-cultural factors, by retrospectively exploring the redundancy and complexity of their experiences. Had the authors reported the unpublished results of their studies instead of summarizing their previous studies, the readers would have benefited more. Innovatively employing retrodictive research agenda, Chapter 11 gives credence to the complexity and dynamicity of class climates in two different groups in a Japanese high school. The authors postulate that CDST “will empower teachers and, in return, their awareness about the complexity of teaching will enlighten us about the realities of teaching in a classroom full of complexity” (p. 187). Informed by directed motivational currents, Chapter 12 emphasizes the pivotal utility of cooperation between teachers-as-co-researchers and researchers, by scrutinizing the applicability of formative experiments as a contextualized way of delving into the dynamic emergence of group-level motivation in an Australian context.

Chapter 13 unpacks how autoethnography and social network analysis pave the solid foundation to unravel the intricate dynamicity of two students concerning their group work, seating positions, and the perception of introvert vs. extrovert features. The author concludes that “learners are indeed people-in-context” (p. 230). Set in the Sweden context, Chapter 14 utilizes introspective and dialogical data to spell out how student-teacher perceptions of teacher identity and contradictory selves emerged over a four-week teaching practicum, mediated by the presence or absence of a mentor teacher in the class. The penultimate chapter draws on the microgenetic approach and frame analysis to investigate language teacher's cognition. The author analyzed the data gained through her interactions with one of her PhD students, concluding that “a context-sensitive analysis of a teacher's tacit and declared belief” (p. 266) enables researchers to figure out the intricacies of interaction. The closing chapter provides an overview of studies informed by CDST and recommends that complexity research should be meaningful and doable for practitioner-researchers.

Although we enjoyed reading each chapter of this volume, if the editors included a chapter on the dynamic nature of boredom in EFL/ESL classes, it would have been insightful as well. Despite the shortcomings, the merits of this compendium are as follows. First, it successfully integrates theory into practice in different chapters. Second, the chapters inform us how such factors as dynamics, timescales, fractals, self-organization, co-adaptation, emergence, etc. can be instantiated through different data collection methods. Thirdly, the chapters eliminate viable illustrations of how complexity research can be conducted, with persuasive evidence of why a complexity viewpoint is fruitful to conceptualize the psychology of language learners and teachers. Therefore, we feel confident to suggest this compendium to language learners, teachers, researchers, and practitioners who are interested in the intersection of complexity and learning.

## Author Contributions

CZ and RZ read this book together and compared their notes on it and agreed to the final version before it is submitted. Then, CZ drafted the first manuscript. RZ revised the language. Both authors contributed to the article and approved the submitted version.

## Conflict of Interest

The authors declare that the research was conducted in the absence of any commercial or financial relationships that could be construed as a potential conflict of interest.

## Publisher's Note

All claims expressed in this article are solely those of the authors and do not necessarily represent those of their affiliated organizations, or those of the publisher, the editors and the reviewers. Any product that may be evaluated in this article, or claim that may be made by its manufacturer, is not guaranteed or endorsed by the publisher.

